# Tracking Healthy People 2020 Internet, Broadband, and Mobile Device Access Goals: An Update Using Data From the Health Information National Trends Survey

**DOI:** 10.2196/13300

**Published:** 2019-06-24

**Authors:** Alexandra J Greenberg-Worisek, Shaheen Kurani, Lila J Finney Rutten, Kelly D Blake, Richard P Moser, Bradford W Hesse

**Affiliations:** 1 Mayo Clinic College of Medicine and Science Rochester, MN United States; 2 National Cancer Institute, National Institutes of Health Rockville, MD United States

**Keywords:** Healthy People 2020, digital divide, internet

## Abstract

**Background:**

As the year 2020 approaches, there is a need to evaluate progress toward the United States government’s Healthy People 2020 (HP2020) health information technology and communication objectives to establish baselines upon which Healthy People 2030 objectives can be based.

**Objective:**

The aim of this study was to use the National Cancer Institute’s (NCI) Health Information National Trends Survey (HINTS) to benchmark progress toward HP2020 goals related to increasing internet access using broadband, and to assess the state of the digital divide for various sociodemographic groups.

**Methods:**

We merged and analyzed data from 8 administrations of HINTS (2003-2017). Descriptive statistics were generated, and predicted marginals were calculated using interaction terms between survey year and selected sociodemographic variables of interest, including age, sex, race and ethnicity, income, education, and geography (rural versus urban), to test for differential change over time.

**Results:**

The number of users having access to the internet increased between 2003 and 2014 (63.15% [3982/6358] to 83.41% [2802/3629]); it remained relatively steady from 2014 to 2017 (81.15% [2533/3283]). Broadband access increased between 2003 and 2011 (from 32.83% [1031/3352] to 77.87% [3375/4405]), but has been declining since (55.93% [1364/2487] in 2017). Access via cellular network increased between 2008 and 2017 (from 6.86% [240/4405] to 65.43% [1436/2489]). Statistically significant disparities in overall internet access were noted in the predicted marginals for age, sex, race and ethnicity, income, and education; for age, sex, income, and geography for broadband access; and for age and sex for cellular network.

**Conclusions:**

The targets set forth in HP2020 were met for overall internet access and for internet access via cellular network; however, the target was not met for internet access via broadband. Furthermore, although the digital divide persisted by sociodemographic characteristics, the magnitude of many disparities in access decreased over time.

## Introduction

The Healthy People initiative sets 10-year objectives for improving the health of Americans nationwide based on the latest scientific evidence [1]. Some Healthy People objectives focus on communication-related objectives including access to information and communication resources such as the internet and mobile devices. These objectives related to technology access speak to their increasing importance in managing health and health care in the United States.

The Office of Disease Prevention and Health Promotion (ODPHP) selects relevant and scientifically rigorous data sources to benchmark progress toward objectives outlined in Healthy People programs. For the Health Communication and Health Information Technology (HC/HIT) objectives related to internet access, ODPHP chose items regularly included in the National Cancer Institute (NCI)’s Health Information National Trends Survey (HINTS), a nationally representative, probability-based survey whose primary aims are to track health behaviors, communication, and technology use [2].

In Healthy People 2020 (HP2020), one of the HC/HIT objectives is to *increase the proportion of individuals with access to the internet* to 75.4%, a 10% improvement over the percentage observed in 2007 (68.5%) [3]. There are 2 additional subobjectives within this broader objective of increasing overall internet access: (1) to increase internet access via broadband by 10% (from 75.6% of those with internet access in 2007 [HP2020 Target: 83.2%]) and (2) to increase internet access via mobile by 10% (from 6.7% of those with internet access in 2007 [HP2020 Target: 7.4%]).

As the Healthy People initiative enters its fourth decade and fourth iteration with the upcoming Healthy People 2030 (HP2030) objectives, it is necessary to reflect upon the HP2020 objectives and assess whether these targets were met and whether these objectives remain relevant in the context of a rapidly evolving communication technology landscape [4]. We analyzed recent HINTS data (2017) to update our previous report on progress toward these HC/HIT objectives and to examine current disparities in access to the internet via broadband and cellular network [5]. In addition, we examine the impact of geography (urban vs rural residence) on internet access via these different connections.

## Methods

### Survey Population and Data Collection

Data from 8 administrations of the NCI’s HINTS were merged for these analyses (N=37,415; Table 1; expanded from Serrano et al [5]). Briefly, HINTS is a national cross-sectional survey of US adults that collects data from the public on a broad range of health and cancer information, communication, attitudes and behaviors, and use of health information technologies. HINTS uses a probability-based sampling frame with a 2-level design in which residential addresses in the United States are sampled, and then 1 adult from each address is randomly selected for participation. In later administrations of HINTS (HINTS 4 and later), efforts were made specifically to oversample for those residing in central Appalachia as well as minority populations. For additional details, please see the corresponding methodology reports for each administration [2,6-8].

**Table 1:** Details of the 8 survey administrations of Health Information National Trends Survey, during 2003-2018 (N=37,415).

Variable	HINTS^a^ 1 (2003)	HINTS 2 (2005)	HINTS 3 (2007-2008)	HINTS 4 Cycle 1 (2011)	HINTS 4 Cycle 2 (2012)	HINTS 4 Cycle 3 (2013)	HINTS 4 Cycle 4 (2014)	HINTS 5 Cycle 1 (2017)
Survey period	Oct 2002-Apr 2003	Feb 2005-Aug 2005	Jan 2008-May 2008	Oct 2011-Jan 2012	Oct 2012-Dec 2012	Sept 2013-Dec 2013	Aug 2014-Nov 2014	Jan 2017-Mar 2017
Respondents (n)	6369	5586	Mail: 3582; RDD^b^: 4092	3959	3630	3185	3677	3335
Survey mode	RDD	RDD	Mail and RDD	Mail	Mail	Mail	Mail	Mail
Response rate (%)	33.1	20.8	Mail: 40.0; RDD: 24.2	36.7	40.0	35.2	34.4	32.4

^a^HINTS: Health Information National Trends Survey.

^b^RDD: random digit dialing.

### Dependent Variables

Our primary outcome variable of interest was internet access; the specific survey item used was *Do you ever go on-line to access the Internet or the World Wide Web, or to send and receive e-mail?* Wording of this item was consistent across survey administrations.

The second survey item of interest was aimed at assessing progress on the HP2020 goals for broadband access. This item was asked in each of the 8 survey administrations; however, wording of this item changed throughout the survey administrations as technology advanced. The wording for HINTS 1-3 was as follows: “When you use the internet at home, do you mainly access it through…cable or satellite modem?” and “…a DSL modem?” These 2 items were combined into 1 variable, to better align with the wording in HINTS 4 [all cycles] and HINTS 5 Cycle 1: “…broadband such as DSL [digital subscriber line], cable, or FiOS [fiber optic service]?”

Finally, the third HP2020 goal examined was for cellular internet access. To examine cellular internet access, the following item was examined: “When you use the Internet do you access it through…a cellular network?” [Yes/No; HINTS 3, HINTS 4 Cycles 1-4, HINTS 5 Cycle 1].

### Independent Variables

Included in the analyses were sociodemographic variables shown to be related to the digital divide (age, sex, race and ethnicity, education, income, and geography) [9-11]. Age was analyzed categorically rather than continuously, as was income. Sex responses were either male or female. Education was categorized as less than high school, high school, some college, and college graduate or higher. Geography was dichotomized into rural and urban using the US Department of Agriculture’s Rural-Urban Continuum Codes (RUCCs). Urban categorization included RUCCs 1-3, which represent metro area counties with greater than 20,000 residents; rural categorization included RUCCs 4-9, which represent nonmetro counties with populations ranging from 2500 to 20,000 [12].

### Statistical Analyses

Analyses were conducted using SAS 9.4 survey procedures to accommodate the complex sampling procedure used and incorporate the jackknife replicate weights. All analyses were weighted to produce population-level point estimates. Descriptive statistics were calculated for all items. In addition, sociodemographic factors were analyzed using logistic regression analyses with predicted marginals to determine whether there existed significant differences in groups for each of the outcome variables. Interaction terms were included between each independent variable and survey year to investigate differential change over survey years.

## Results

### Overview

Although respondent characteristics varied slightly across survey administrations, the weighted respondent characteristics closely reflected those of the US Census for each respective iteration. As expected in most mailed survey studies, most respondents were female, aged 18 to 34, non-Hispanic white, had at least some college education, and had a household income of US $75,000 or more annually (data not shown; available on the website [13]).

### Internet Access

**Figure 1:**
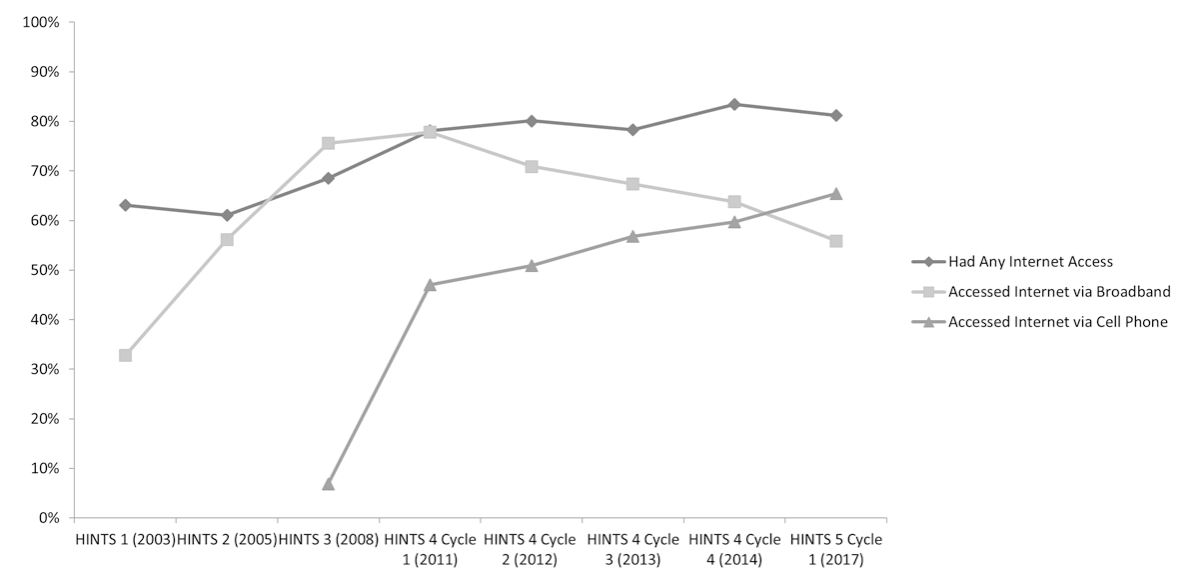
Percentage of US adult population with access to internet (out of all respondents), access to internet via broadband (out of respondents with internet access), and access to internet via cell phone (out of respondents with internet access), Health Information National Trends Survey (HINTS) 2003-2017. Survey question on accessing the internet through a cellular network not included in HINTS 1 (2003) or HINTS 2 (2005) survey administrations.

Internet access increased overall between 2003 and 2014 (20.3 percentage points, from 63.15% [3982/6358] to 83.41% [2802/3629]), with some variation along the way; however, the percentage of those with internet access remained relatively steady from 2014 to 2017 (Figure 1).

In the multivariable regression model, all of the sociodemographic variables examined showed statistical significance between groups after adjusting for survey year; however, the magnitude of differences was not significant across all groups within each variable (Multimedia Appendix 1). Briefly, men had a 0.74-fold decreased odds of having internet access as compared with women (95% CI 0.67-0.83). All age categories 35 years and above had significantly lower odds of having internet access compared with those in the 18 to 34 years old referent group; these odds ranged from 0.42 in the 35 to 49 year old age group (95% CI 0.35-0.51) to 0.04 in the 75+ age group (95% CI 0.03-0.05). Every race and ethnicity group was significantly less likely to have internet access compared with the non-Hispanic white referent group; Hispanic respondents had a 0.37 odds (95% CI 0.31-0.43), non-Hispanic Black had 0.51 odds (95% CI 0.44-0.59), and non-Hispanic Other had 0.45 (95% CI 0.34-0.61).

Those with higher levels of education had significantly higher odds of having internet access versus those with less than a high school education; for example, college graduates had an increased odds of having internet access compared with those who did not graduate from high school (odds ratio [OR] 9.44; 95% CI 7.65-11.65). Similarly, those with higher annual incomes had increased odds compared with those with lower annual incomes (ie, those with US $75,000 or higher annual income had 6.67-fold increased odds of having internet access compared with those with less than US $20,000 per year income [95% CI 5.54, 8.04]). Finally, those living in rural areas had reduced odds of having internet access compared with those residing in urban areas (OR 0.75; 95% CI 0.67-0.84).

**Figure 2:**
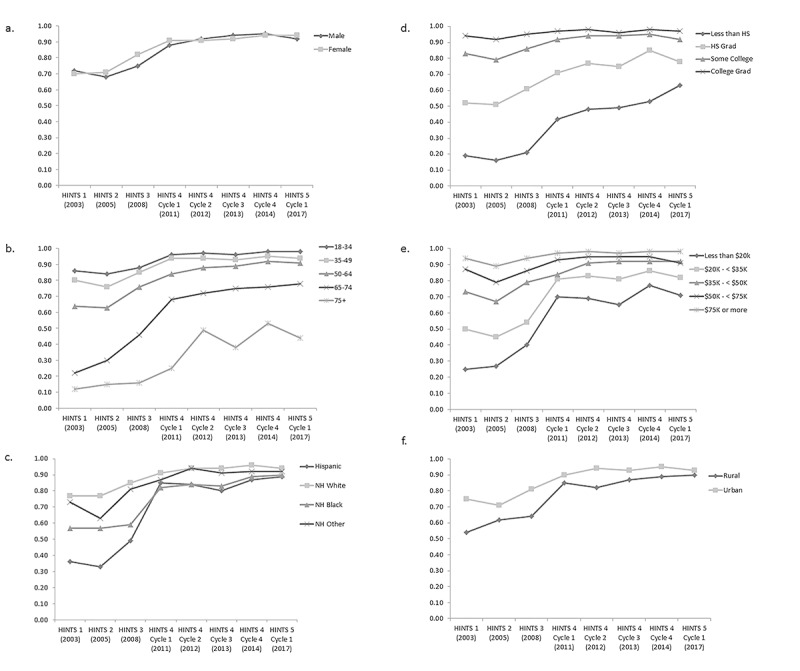
Trends of having internet access based on responses from the National Cancer Institute’s Health Information National Trends Survey administrations between 2003 and 2017. (a) Predicted marginals by sex. (b) Predicted marginals by age. (c) Predicted marginals by race and ethnicity. (d) Predicted marginals by education. (e) Predicted marginals by income. (f) Predicted marginal by geography. All models adjusted for sex, age, race and ethnicity, education, income, and geography. NH: non-Hispanic.

Although all sociodemographic variables examined were statistically significant in our multivariable logistic regression, age category, sex, education, income, and race and ethnicity had significant interactions with survey year (Figure 2). The interaction between survey year and geography had borderline statistical significance (*P*=.07). For most groups examined within each of these variables, the overall trend was toward increasing internet access over time.

### Internet Access via Broadband

Broadband access increased overall by 42.8 percentage points from 2003 to 2011 (from 32.83% [1031/3352] to 77.87% [3375/4405]); however, it has continually decreased since then (to 55.93% [1364/2487] in 2017, 21.9 percentage points; Figure 1). It should be noted that broadband access from the perspective of the HINTS survey emphasizes wired access to the home (eg, through FiOS, cable, and DSL). It will be distinguished for the purposes of this paper from cellular access to the internet as provided through mobile phone technologies and mobile data plans.

In the multivariable logistic regression model of having broadband access among those with internet access, all sociodemographic variables were again statistically significant after adjusting for survey year; however, the magnitude of some of these differences was greatly reduced (Multimedia Appendix 2). Although most trends within variables remained the same, there were a few notable changes. First, males who reported having internet access had a 1.67-fold increased odds of having broadband access as compared with their female counterparts (95% CI 1.51-1.85). The only significant difference among race and ethnicity groups was the reduced odds of having broadband among Hispanic respondents reporting having internet access as compared with non-Hispanic white respondents (OR 0.77; 95% CI 0.63-0.94); there was no significant difference for non-Hispanic blacks and non-Hispanic other compared with non-Hispanic whites, suggesting they are no more or less likely to have broadband internet access. Within education categories, there was no significant difference between high school graduates as compared with nonhigh school graduates (OR 1.33; 95% CI 0.93-1.91); differences remained significant for those who had some college or who were college graduates, compared with those with less than a high school diploma (OR 1.84; 95% CI 1.32-2.55 for those with some college and OR 1.97; 95% CI 1.42-2.74 for those with college graduates). Similarly, differences were no longer statistically significant between those with annual incomes of US $20,000-50,000 compared with those of less than US $20,000 per year; they remained significantly different for higher income levels (OR 1.45, 95% CI 1.14-1.84 for US $50,000 to < US $75,000; OR 1.85, 95% CI 1.55-2.21 for > US $75,000; Multimedia Appendix 2).

**Figure 3:**
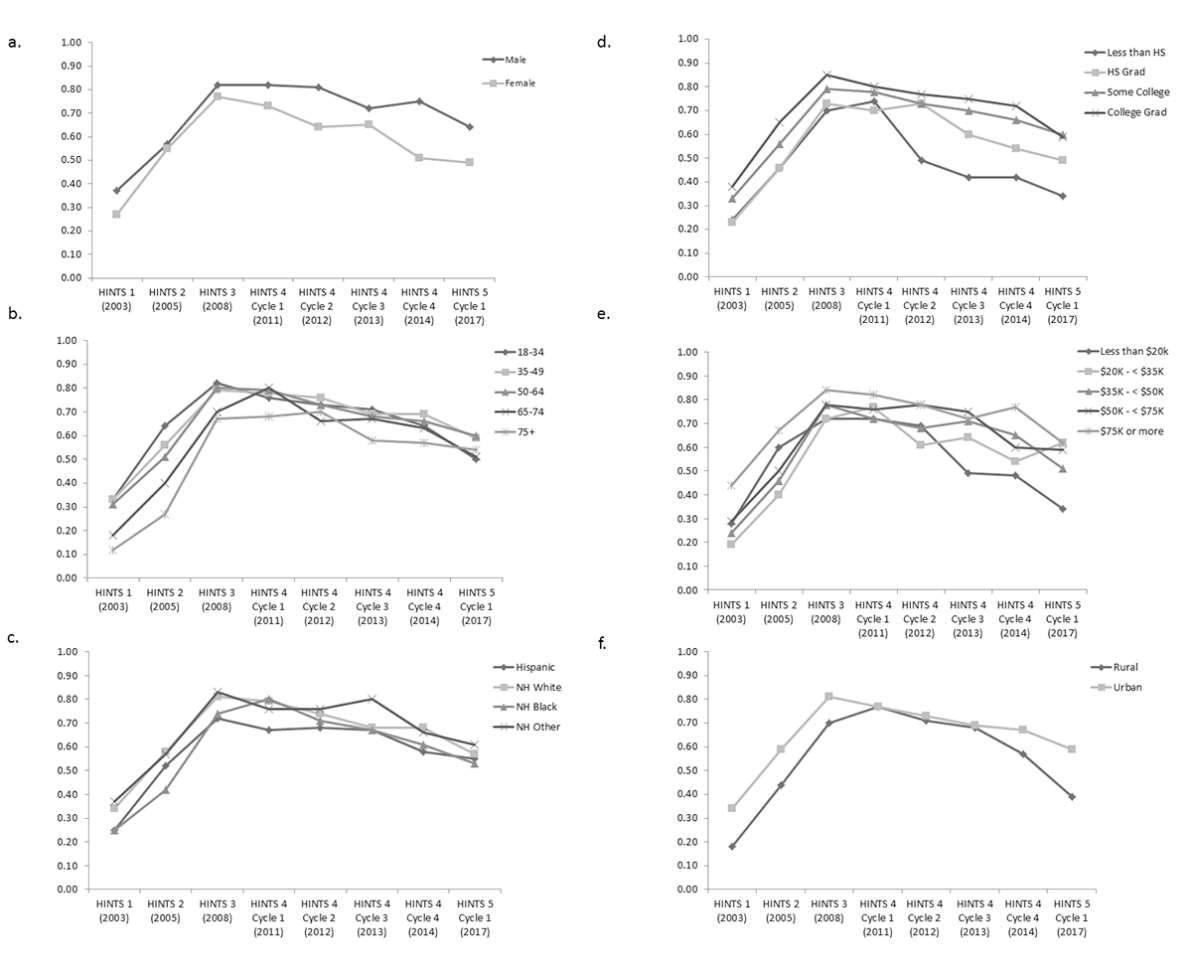
Trends of having internet access via broadband based on responses from the National Cancer Institute’s Health Information National Trends Survey administrations between 2003 and 2017. (a) Predicted marginals by sex. (b) Predicted marginals by age. (c) Predicted marginals by race and ethnicity. (d) Predicted marginals by education. (e) Predicted marginals by income. (f) Predicted marginal by geography. All models adjusted for sex, age, race and ethnicity, education, income, and geography. NH: non-Hispanic.

Although all sociodemographic variables examined were statistically significant in our multivariable logistic regression, only age category, sex, income, and geography showed statistically significant interactions with survey year (Figure 3), suggesting that access changed over time for these groups. For most groups examined within each of the sociodemographic variables, the overall trend was toward increasing internet access via broadband until 2011, with a subsequent decrease after that year.

### Internet Access via Cellular Network

Internet access via cellular network increased from 2008 to 2017 (58.5 percentage points, from 6.86% [240/4405] to 65.43% [1436/2489]); the greatest increase was between 2008 and 2011 (40.1 percentage points, from 6.86% [240/4405] to 47.01% [1128/2861]; Figure 1). This finding presents a contrasting trend to the recent decline in internet access through traditional landline, fiber optic, or cable broadband to the home.

Most of the sociodemographic variables within our multivariable model were statistically significant after adjusting for survey year (Table 2). No significant difference was found between males and females in accessing the internet via cellular networks, nor for rural versus urban residents. There was no significant difference in having internet access via cellular network for Hispanics and non-Hispanic blacks compared with non-Hispanic whites. Similarly, there was no statistically significant difference between educational groups of high school graduates and above compared with those who had less than a high school education. Odds of accessing the internet via cellular network decreased with increasing age in a statistically significant manner for each age category above 35 years of age, compared with those 18 to 34 years of age. Differences were not significant between those with annual incomes of US $20,000 to 50,000 compared with those of less than US $20,000 per year, but again remained significantly different for higher income levels (Table 2).

**Table 2:** Weighted multivariate logistic regression model of predictors of having internet access via mobile phone among those who reported having internet access. Data from the National Cancer Institute’s Health Information National Trends Survey (HINTS) administrations between 2008 and 2017 (n=14,794).

Variable		Predictors of internet access via cell phone				
		Odds ratio (95% CI)	Beta coefficient	SE beta	Adjusted Wald F	*P* value
**Sex**					1.32	.252
	Female	Ref^a^	Ref	Ref		
	Male	1.08 (0.95, 1.22)	0.7	0.06		
**Age**					166.15	<.001
	18-34	Ref	Ref	Ref		
	35-49	0.43 (0.36-0.51)	–0.84	0.09		
	50-64	0.20 (0.17-0.24)	–1.61	0.10		
	65-74	0.08 (0.06-0.10)	–2.52	0.11		
	>75	0.04 (0.03-0.05)	–3.24	0.16		
**Race and ethnicity**					4.07	.008
	Non-Hispanic White	Ref	Ref	Ref		
	Hispanic	1.25 (1.00-1.56)	0.23	0.11		
	Non-Hispanic Black	1.39 (1.09-1.77)	0.33	0.12		
	Non-Hispanic Other	0.83 (0.63-1.10)	–0.18	0.14		
**Education**					5.26	.002
	Less than high school	Ref	Ref	Ref		
	High school graduate	1.03 (0.65-1.64)	0.03	0.24		
	Some college	1.42 (0.89-2.27)	0.35	0.24		
	College graduate	1.47 (0.93-2.31)	0.38	0.23		
**Income (US $)**					14.06	<.001
	<$20,000	Ref	Ref	Ref		
	$20,000 to <$35,000	0.98 (0.73-1.30)	–0.02	0.15		
	$35,000 to <$50,000	1.07 (0.83-1.39)	0.07	0.13		
	$50,000 to <$75,000	1.33 (1.04-1.70)	0.28	0.13		
	$75,000 +	1.92 (1.50-2.46)	0.65	0.12		
**Geography**					4.60	.033
	Urban	Ref	Ref	Ref		
	Rural	0.80 (0.65-0.98)	–0.23	0.11		
**HINTS ^b^** **Survey Year**					126.77	<.001
	HINTS 3 (2008)	Ref	Ref	Ref		
	HINTS 4 Cycle 1 (2011)	17.86 (13.17-24.21)	2.88	0.15		
	HINTS 4 Cycle 2 (2012)	21.59 (16.06-29.02)	3.07	0.15		
	HINTS 4 Cycle 3 (2013)	29.45 (21.32-40.69)	3.38	0.16		
	HINTS 4 Cycle 4 (2014)	30.45 (22.24-41.69)	3.42	0.16		
	HINTS 5 Cycle 1 (2017)	51.31 (37.54-70.11)	3.94	0.16		

^a^Ref: reference group.

^b^HINTS: Health Information National Trends Survey.

**Figure 4:**
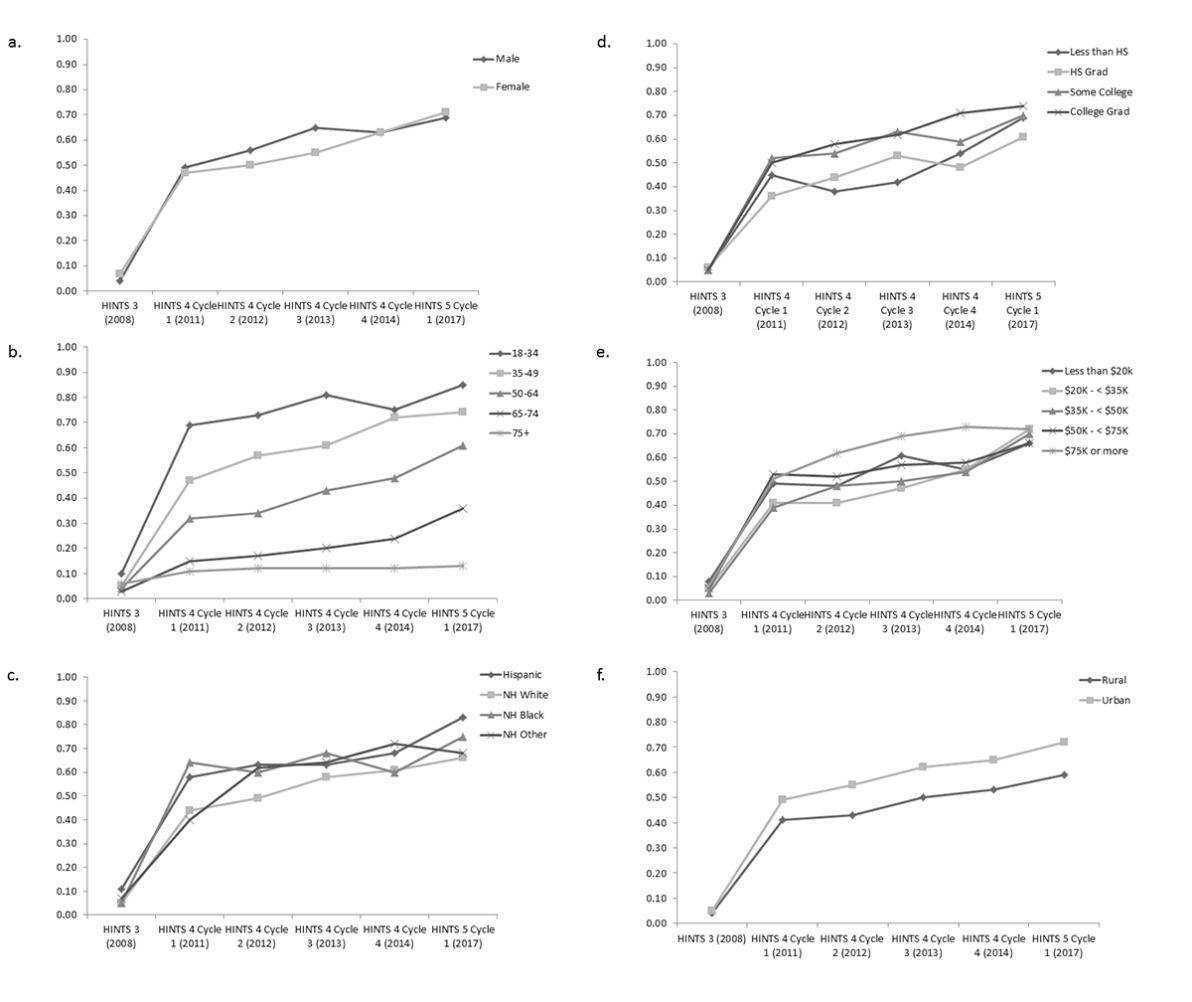
Trends of having internet access via cell phone/mobile based on responses from the National Cancer Institute’s Health Information National Trends Survey administrations between 2008 and 2017. (a) Predicted marginals by sex. (b) Predicted marginals by age. (c) Predicted marginals by race and ethnicity. (d) Predicted marginals by education. (e) Predicted marginals by income. (f) Predicted marginal by geography. All models adjusted for sex, age, race and ethnicity, education, income, and geography. NH: non-Hispanic.

Although most sociodemographic variables examined were statistically significant, only age category and sex had significant interactions with survey year (Figure 4), suggesting that mobile technology may have helped or be helping bridge the digital divide across income, race and ethnicity, education, and geography over time [14,15].

## Discussion

### Principal Findings

The results presented in this study provide a timely update on progress toward the HP2020 goals related to access to the internet during a time of rapid changes in communication and information technology. The HP2020 objective to increase internet access overall for Americans by 10% from the 2007 baseline percentage of 68.5% (HP2020 Target: 75.4%) [3] was surpassed in 2011 (78.1%), as noted in our 2016 report; it has continued to remain relatively steady since the previous report (81.2% in 2017) [5]. The HP2020 objective to increase internet access via broadband by 10% from the baseline percentage of 75.6% in 2007 (HP2020 Target: 83.2%) was never reached, as it peaked at 77.8% in 2011 and then steadily declined. As stated earlier, we believe that this may be due to the increasing shift toward internet access via cellular network, as the data presented here demonstrate. The HP2020 objective to increase internet access via cellular network by 10% from the baseline of 6.7% in 2007 (HP2020 Target: 7.4%) was greatly surpassed, with 65.4% of individuals in 2017 reporting internet access via cellular network.

Overall, internet access has remained relatively stable over the last 5 years; however, contrasting trends in traditional wired access versus cellular access illustrate an important nuance over the ways in which populations access the internet that was not yet obvious in the previous publications [4,5]. Broadband access to the home offers always-on, high-speed capacity to search for health information, review or download data from a personal health record, to order medications, and so on. The rise of cellular access provides patients with an always-on, always present capacity. It should be noted, then, that the steady state of internet access reflects divergence across channels, with some capabilities common to both (eg, internet-based searching) and with some capabilities ideally suited for one over the other. For example, some websites that contain detailed information may not be easily viewed on a mobile phone; however, the use of a tablet via cellular network may allow such information to be accessed fully. Of the variables examined, only age has statistically significant interactions over time across internet access overall, internet access via broadband, and internet access via cellular network. This suggests that the digital divide by age persists, wherein older adults are less likely to report overall internet access than those aged 18 to 34.

That disparities in overall internet access and via broadband exist independent of survey administration indicates that a digital divide persists for many; however, our predicted marginals reported here indicate that the magnitude of these divides may be narrowing for some groups [16]. These findings are consistent with studies that show that the introduction of the smartphone in 2007 has helped to narrow gaps in internet access for many over the past ten years [17-19]. That, coupled with the decreasing cost of such technology and data plans, means that internet access via cellular network is within reach for a broader proportion of Americans. Specific groups may benefit from targeted interventions to increase access to and acceptance of the internet, such as members of older age groups who face greater health challenges and may therefore benefit from access to health information and communication technologies [14,20].

Although a significant difference was observed between rural and urban residents in overall internet access and in accessing the internet via broadband among those who reported having internet access, no difference was seen in internet access via cellular network among those reporting having internet access. We hypothesize that this may be explained in part due to the fact that there are fewer infrastructural barriers for expanding internet access via cellular network to rural areas than there are for expanding internet access via broadband, as well as due to the reduction in broadband expansion after 2014 reported by the Federal Communications Commission [21]. Such information can be used to help public health planners to provide more effective mobile health and telehealth interventions in rural areas, especially given that rural residents are equally likely to report that their providers utilize electronic health records [22].

Strengths of this study include the use of HINTS, which uses a scientifically rigorous probability-based sample and is nationally representative of the US adult population and oversamples for underrepresented populations. HINTS is meant to assess trends over time, with many core items having been collected repeatedly over the past 15 years; this allows for tracking of key metrics related to the HP2020 objectives. In addition, the weighting paradigm allows for weighted percent estimates reflective of the US population. Limitations include those associated with cross-sectional surveys, such as the inability to determine cause and effect. A second lies in the limitations of self-reported measures; that is, individuals may complete the survey with satisficing answers, not correctly recall information needed to answer the items, or leave items blank due to their sensitive nature. A final limitation is that response rates are lower than one would find in a prospective study of those actively engaged with the health care system.

### Conclusions

The objectives and targets set forth by the HP2020 initiative for overall internet access and internet access via cellular network were met and, in the case of cellular network access, greatly surpassed. In addition, this study found that the digital divide still exists for many; however, the magnitude of these gaps is narrowing for several groups. However, internet access via traditional broadband delivery to the home began to decline before meeting the HP2020 target value. We believe that this is reflective of the rapidly changing technology landscape; that is, the objectives included in HP2020 were very specific to the means of accessing the internet available at the time the objectives were initially written, and the adoption of technology—and technology itself—evolved far more quickly than could have been anticipated. In creating objectives for HP2030, it could be beneficial to use wording broad enough to accommodate changes in modality for accessing the internet.

## Appendix

Multimedia Appendix 1 Weighted multivariate logistic regression model of predictors of having internet access. Data from the National Cancer Institute’s Health Information National Trends Survey administrations between 2003 and 2017 (n=30,150).

Multimedia Appendix 2 Weighted multivariate logistic regression model of predictors of having broadband internet access among those who reported having internet access. Data from the National Cancer Institute’s Health Information National Trends Survey administrations between 2003 and 2017 (n=20,150).

## Abbreviations

DSL: digital subscriber line
FiOS: fiber optic service
HC/HIT: Health Communication and Health Information Technology
HINTS: Health Information National Trends Survey
HP2020: Healthy People 2020
NCI: National Cancer Institute
ODPHP: Office of Disease Prevention and Health Promotion
OR: odds ratio
RUCCs: Rural-Urban Continuum Codes
