# Correction: Tracking Healthy People 2020 Internet, Broadband, and Mobile Device Access Goals: An Update Using Data From the Health Information National Trends Survey

**DOI:** 10.2196/39712

**Published:** 2022-05-26

**Authors:** Alexandra J Greenberg-Worisek, Shaheen Kurani, Lila J Finney Rutten, Kelly D Blake, Richard P Moser, Bradford W Hesse

**Affiliations:** 1 Mayo Clinic College of Medicine and Science Rochester, MN United States; 2 National Cancer Institute, National Institutes of Health Rockville, MD United States

In “Tracking Healthy People 2020 Internet, Broadband, and Mobile Device Access Goals: An Update Using Data From the Health Information National Trends Survey” (J Med Internet Res 2019;21(6):e13300) the authors made the following updates.

The authors were notified of data errors in two of the Health Information National Trends Survey (HINTS) cycle datasets (HINTS 4, Cycle 3 and HINTS 4, Cycle 4); the errors were in the weights provided for use in the analysis of these data [1].

These weights primarily affected logistic regression analyses, reported in Table 2 of the originally published article. This previous version of Table 2 is in Multimedia Appendix 1. Following the HINTS error notice [1], the authors reran the logistic regression analyses. The corrected version of the article includes the following updated Table 2.

In rerunning this analysis, only one difference was found that resulted in changes in the conclusion. Namely, geography is significant, with those in rural settings having significantly lower odds of having internet access via a mobile phone compared to their urban counterparts (OR = 0.80; 95% CI: 0.65-0.98; *P*=.033). This finding was not reported as significant in the original analysis, due to the above-mentioned error in the HINTS data sets [1]. All other conclusions remain consistent with those reported in the original publication.

In the section “Internet Access via Cellular Network” in the *Results*, the first sentence in the second paragraph originally read as follows:

Most of the sociodemographic variables within our multivariable model were statistically significant after adjusting for survey year, save for geography (Table 2).

It has been corrected as follows:

Most of the sociodemographic variables within our multivariable model were statistically significant after adjusting for survey year (Table 2).

**Table 2:** Weighted multivariate logistic regression model of predictors of having internet access via mobile phone among those who reported having internet access. Data from the National Cancer Institute’s Health Information National Trends Survey (HINTS) administrations between 2008 and 2017 (n=14,794).

Variable		Predictors of internet access via cell phone				
		Odds ratio (95% CI)	Beta coefficient	SE beta	Adjusted Wald F	*P* value
**Sex**					1.32	.252
	Female	Ref^a^	Ref	Ref		
	Male	1.08 (0.95, 1.22)	0.7	0.06		
**Age**					166.15	<.001
	18-34	Ref	Ref	Ref		
	35-49	0.43 (0.36-0.51)	–0.84	0.09		
	50-64	0.20 (0.17-0.24)	–1.61	0.10		
	65-74	0.08 (0.06-0.10)	–2.52	0.11		
	>75	0.04 (0.03-0.05)	–3.24	0.16		
**Race and ethnicity**					4.07	.008
	Non-Hispanic White	Ref	Ref	Ref		
	Hispanic	1.25 (1.00-1.56)	0.23	0.11		
	Non-Hispanic Black	1.39 (1.09-1.77)	0.33	0.12		
	Non-Hispanic Other	0.83 (0.63-1.10)	–0.18	0.14		
**Education**					5.26	.002
	Less than high school	Ref	Ref	Ref		
	High school graduate	1.03 (0.65-1.64)	0.03	0.24		
	Some college	1.42 (0.89-2.27)	0.35	0.24		
	College graduate	1.47 (0.93-2.31)	0.38	0.23		
**Income (US $)**					14.06	<.001
	<$20,000	Ref	Ref	Ref		
	$20,000 to <$35,000	0.98 (0.73-1.30)	–0.02	0.15		
	$35,000 to <$50,000	1.07 (0.83-1.39)	0.07	0.13		
	$50,000 to <$75,000	1.33 (1.04-1.70)	0.28	0.13		
	$75,000 +	1.92 (1.50-2.46)	0.65	0.12		
**Geography**					4.60	.033
	Urban	Ref	Ref	Ref		
	Rural	0.80 (0.65-0.98)	–0.23	0.11		
**HINTS ^b^** **Survey Year**					126.77	<.001
	HINTS 3 (2008)	Ref	Ref	Ref		
	HINTS 4 Cycle 1 (2011)	17.86 (13.17-24.21)	2.88	0.15		
	HINTS 4 Cycle 2 (2012)	21.59 (16.06-29.02)	3.07	0.15		
	HINTS 4 Cycle 3 (2013)	29.45 (21.32-40.69)	3.38	0.16		
	HINTS 4 Cycle 4 (2014)	30.45 (22.24-41.69)	3.42	0.16		
	HINTS 5 Cycle 1 (2017)	51.31 (37.54-70.11)	3.94	0.16		

^a^Ref: reference group.

^b^HINTS: Health Information National Trends Survey.

In addition, the corresponding author's email address has been changed from *greenberg.alexandra@mayo.edu* to *worisek.alexandra@gmail.com*, as the author is no longer affiliated with Mayo Clinic College of Medicine and Science.

The correction will appear in the online version of the paper on the JMIR Publications website on May 26, 2022 together with the publication of this correction notice. Because this was made after submission to PubMed, PubMed Central, and other full-text repositories, the corrected article has also been resubmitted to those repositories.

## Appendix

Multimedia Appendix 1 Originally published Table 2.

## Abbreviations

HINTS: Health Information National Trends Survey
