# Supplementary material for: Tracking Healthy People 2020 Internet, Broadband, and Mobile Device Access Goals: An Update Using Data From the Health Information National Trends Survey
Source: J Med Internet Res. 2019 Jun 24;21(6):e13300. doi: 10.2196/13300 (PMC6613328; doi:10.2196/13300)
Supplement: Multimedia Appendix 2 [file jmir_v21i6e13300_app2.docx]

Multimedia Appendix 2**.** Weighted multivariate logistic regression model of predictors of having broadband internet access among those who reported having internet access. Data from the National Cancer Institute’s Health Information National Trends Survey administrations between 2003 and 2017 (n=20,150). “—” indicates “Not applicable.” “Ref” indicates reference group.

|  |  | Predictors of broadband internet access | | | | |
| --- | --- | --- | --- | --- | --- | --- |
|  |  | Odds ratio  (95% CI) | Beta coefficient | SE beta | Adjusted Wald F | *P value* |
|  | | | | | | |
| **Sex** |  | — | — | — | 101.18 | <.001 |
|  | Female | Ref | Ref | Ref | — | — |
|  | Male | 1.67 (1.51-1.85) | 0.51 | 0.05 | — | — |
| **Age** |  | — | — | — | 19.23 | <.001 |
|  | 18-34 | Ref | Ref | Ref | — | — |
|  | 35-49 | 0.96 (0.82-1.12) | –0.04 | 0.08 | — | — |
|  | 50-64 | 0.90 (0.77-1.05) | –0.10 | 0.08 | — | — |
|  | 65-74 | 0.72 (0.61-0.85) | –0.32 | 0.08 | — | — |
|  | >75 | 0.65 (0.49-0.87) | –0.43 | 0.14 | — | — |
| **Race and ethnicity** |  | — | — | — | 6.79 | 0.079 |
|  | NH White | Ref | Ref | Ref | — | — |
|  | Hispanic | 0.77 (0.63-0.94) | –0.26 | 0.10 | — | — |
|  | NH Black | 0.92 (0.77-1.10) | –0.09 | 0.09 | — | — |
|  | NH Other | 1.01 (0.83-1.23) | 0.01 | 0.10 | — | — |
| **Education** |  | — | — | — | 31.76 | <.001 |
|  | Less than high school | Ref | Ref | Ref | — | — |
|  | High school graduate | 1.33 (0.93-1.91) | 0.28 | 0.18 | — | — |
|  | Some college | 1.84 (1.32-2.55) | 0.61 | 0.16 | — | — |
|  | College graduate | 1.97 (1.42-2.74) | 0.68 | 0.16 | — | — |
| **Income (US $)** |  | — | — | — | 74.52 | <.001 |
|  | <$20,000 | Ref | Ref | Ref | — | — |
|  | $20,000 to <$35,000 | 1.19 (0.93-1.53) | 0.18 | 0.12 | — | — |
|  | $35,000 to <$50,000 | 1.31 (1.0-1.71) | 0.27 | 0.13 | — | — |
|  | $50,000 to <$75,000 | 1.45 (1.14-1.84) | 0.37 | 0.12 | — | — |
|  | $75,000 + | 1.85 (1.55-2.21) | 0.61 | 0.09 | — | — |
| **Geography** |  | — | — | — | 13.65 | <.001 |
|  | Urban | Ref | Ref | Ref | — | — |
|  | Rural | 0.75 (0.64-0.87) | –0.29 | 0.08 | — | — |
| **HINTS Survey Year** |  | — | — | — | 1039.89 | <.001 |
|  | HINTS 1 (2003) | Ref | Ref | Ref | — | — |
|  | HINTS 2 (2005) | 2.72 (2.35-3.15) | 1.0 | 0.07 | — | — |
|  | HINTS 3 (2008) | 8.36 (7.08-9.87) | 2.12 | 0.08 | — | — |
|  | HINTS 4 Cycle 1 (2011) | 7.93 (6.47-9.72) | 2.07 | 0.10 | — | — |
|  | HINTS 4 Cycle 2 (2012) | 5.98 (4.92-7.26) | 1.78 | 0.08 | — | — |
|  | HINTS 4 Cycle 3 (2013) | 4.88 (4.02-5.91) | 1.58 | 0.10 | — | — |
|  | HINTS 4 Cycle 4 (2014) | 4.18 (3.47-5.03) | 1.43 | 0.09 | — | — |
|  | HINTS 5 Cycle 1 (2017) | 2.82 (2.32-3.43) | 1.04 | 0.10 | — | — |
