# Supplementary figures and images for: Correction: Tracking Healthy People 2020 Internet, Broadband, and Mobile Device Access Goals: An Update Using Data From the Health Information National Trends Survey
Source: J Med Internet Res. 2022 May 26;24(5):e39712. doi: 10.2196/39712 (PMC9185341; doi:10.2196/39712)

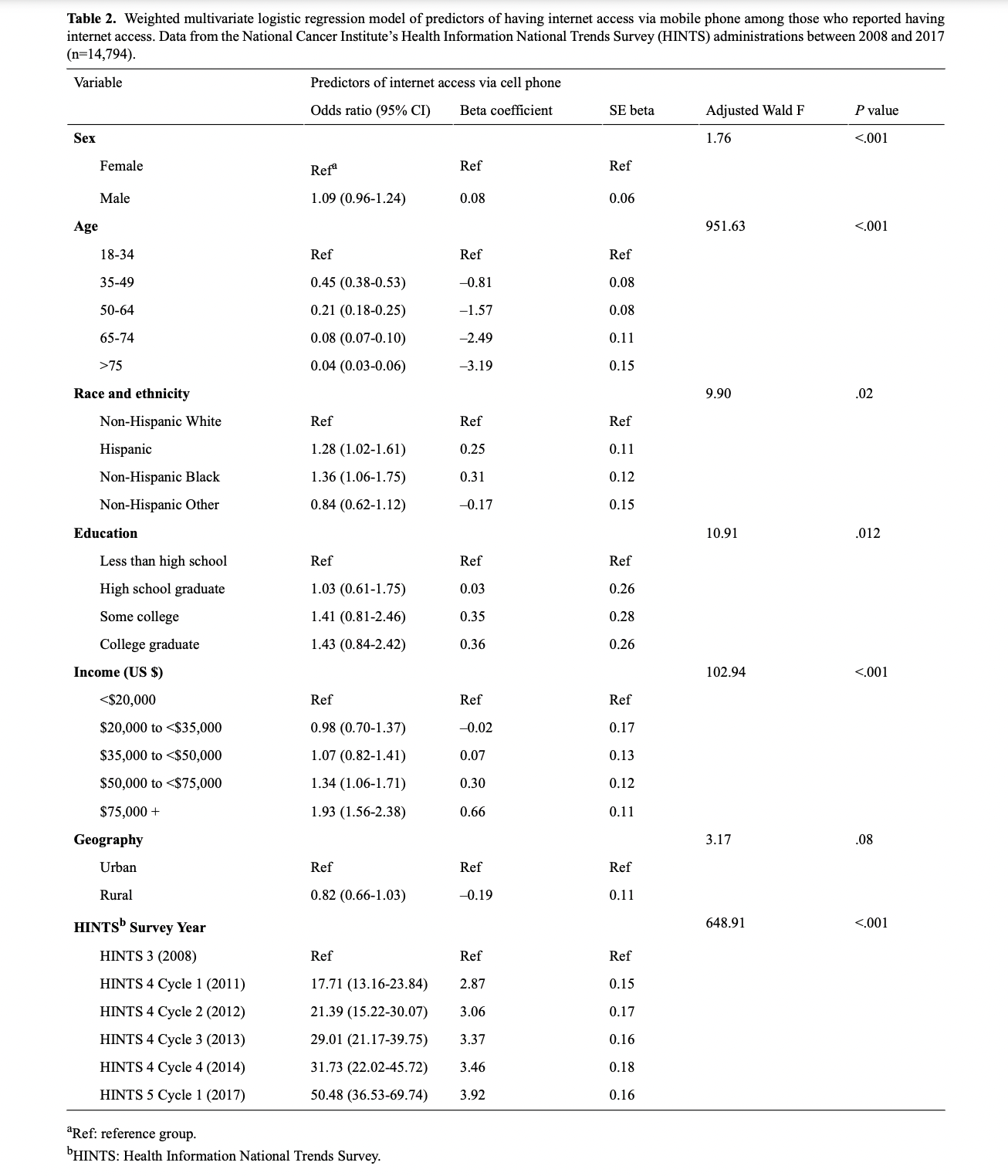

Supplement: Multimedia Appendix 1 [file jmir_v24i5e39712_app1.png]
